# The Health Risk Assessment of Essential Elemental Impurities (Cu, Mn and Zn) Through the Dermal Exposure of Herbal Ointment Extracted from Marjoram Herb (*Majoranae herbae extractum*)

**DOI:** 10.1007/s12011-021-02842-8

**Published:** 2021-07-31

**Authors:** Kamil Jurowski, Maria Fołta, Barbara Tatar, Mehmet Berkoz, Mirosław Krośniak

**Affiliations:** 1 Institute of Medical Studies, Medical College, Rzeszów University, Aleja Majora Wacława Kopisto 2a, 35-959 Rzeszów, Poland; 2 Department of Food Chemistry and Nutrition, Medical College, Jagiellonian University, Medyczna 9, 30-688 Kraków, Poland; 3 Department of Biochemistry, Faculty of Pharmacy, Van Yuzuncu Yil University, 65080 Van, Turkey

**Keywords:** Marjoram herb extract, Essential elemental impurities, Herbal medicinal product, Toxicological risk assessment

## Abstract

Essential elements like Cu, Mn and Zn are extremely important for herbs’ growth and physiological functions; however, from a toxicological point of view, the exposure of these elements (as essential elemental impurities) can exhibit potential harmful effects for patients. In Europe, very popular are ointments with Marjoram herb extract (*Majoranae herbae extractum*) as herbal medicinal products for adjunctively in rhinitis (runny nose). Based on posology of ointments with Marjoram herb extract, the exposure to these elemental impurities may be high during long-term use. Hence, the aim of this article is the health risk assessment of essential elemental impurities (Cu, Mn and Zn) through the dermal exposure of ointments with Marjoram herb extract (*Majoranae herbae extractum*) as herbal medicinal products applied adjunctively in rhinitis available in Polish pharmacies. The investigated essential elements were determined by well-validated methodology (*R* > 0.997, recoveries, LOD and LOQ values were acceptable) based on flame atomic absorption spectrometry (FAAS). Our results indicated that all analysed herbal medicinal products with Marjoram herb extract available in Polish pharmacies contain relatively low levels of essential element impurities, i.e. Cu (0.14–0.49 mg/kg), Mn (0.31–2.57 mg/kg) and Zn (0.73–3.19 mg/kg). The estimated exposure of the investigated elemental impurities confirms the safety of all products. To the best of our knowledge, the study about Cu, Mn and Zn contents in HMPs with *Majoranae herbae extractum* is described for the first time. The applied methodology and results are extremely important from regulatory toxicology point of view due to ICH Q3D elemental impurity guideline for pharmaceuticals.

**Supplementary Information:**

The online version contains supplementary material available at 10.1007/s12011-021-02842-8.

## Introduction

*Origanum majorana* L., herba (Marjoram) is an active pharmaceutical ingredient (API) in herbal medicinal products (HMPs)/phytotherapeutics used as a home remedy for adjunctively in rhinitis (runny nose). Usually, this kind of HMPs exists in semi-solid dosage forms for cutaneous use and are applied for relief of irritated skin around the nostrils [1]. The leaves of *O. majorana* L., herba has been traditionally used for the treatment of gastrointestinal disturbances, cough and bronchial diseases [2, 3]. However, the most important indication is rhinitis (runny nose). Based on posology, a small amount of the preparation should be spread around the nostrils, 2–4 times daily [1]. Hence, from toxicological point of view, there is a potential health risk related to dermal exposure. In this situation, potential health risks may be associated with elemental impurities (EIs). The appropriate control of EIs is currently required for quality assurance for the pharmaceutical industry. However, there is a lack of scientific original articles; there are only a few articles about this important issue [4–7]. Based on posology of ointments with Marjoram herb extract (*Majoranae herbae extractum*) mentioned earlier, exposure to EIs may be high during long-term use. From this point of view, essential EIs can be a very interesting problem and challenge. It is well-known that all herbs require essential elements for physiological functioning and growth [8]. It should be mentioned that essential elements cannot be synthesised by the plants itself; hence, uptake of these kind of elements is crucial [9]. These essential elements (especially Cu, Mn and Zn) are extremely important; however, from a toxicological point of view, the excess of these elements (as essential EIs) can exhibit potential harmful effects for patients [10]. For example, Cu has been recorded and shown to cause problems only under certain specific conditions, notably genetic disorders such as Wilson disease [5]. Additionally, the symptoms of Mn toxicity can result as manganism (a permanent neurological disorder) [5]. On the other hand, chronically high Zn intake can result in severe neurological diseases attributable to copper deficiency as the results of antagonism of both elements [5]. Hence, the aim of our work was the toxicological risk assessment of Cu, Mn and Zn as essential EIs in ointments with Marjoram herb extract (*Majoranae herbae extractum*) applied adjunctively in rhinitis. For this purpose, the levels of Cu, Mn and Zn in samples of HMPs available in Polish pharmacies were determined by atomic absorption spectrometry using flame atomisation (FAAS). To the best of our knowledge, the study about Cu, Mn and Zn contents in HMPs with *Majoranae herbae extractum* is described for the first time.

## Materials and Methods

### Samples

All available in Poland (*n* = 5), ointments with Marjoram herb extract (*Majoranae herbae extractum*) as herbal medicinal products applied adjunctively in rhinitis (runny nose) were investigated. The choice of HMPs was justified by the fact that this kind of pharmaceutical products is very popular in Poland, especially among young children and seniors. It should be underlined that ointments with Marjoram herb extract are monocomponent herbal medicinal products; hence, there is exclusion of other sources of EIs.

All traditional pharmaceutical products were collected from local pharmacies situated in the Lesser Poland Voivodeship (Kraków, Niepołomice, Bochnia, Wieliczka) in 2021. All the investigated samples were pharmaceutical product of individual manufacturers. To maintain the highest methodological standards, each sample was coded (A, B and so on). The short characteristics of the analysed samples was described in Table 1.

**Table 1:** The short characterisation of investigated herbal medicinal products with Marjoram herb extract available in Polish pharmacies

Sample		Main herbal component	Herbal preparation	Licence
No	Code			
1	A	*Majoranae herbae extractum*	*Extract (ratio of herbal substance to extraction solvent 1:5), extraction solvents: ethanol 96% v/v and white petroleum jelly*	IL-2800/LN
2	B			20,902
3	C			IL-3036/LN
4	D			IL-2601/LN
5	E			IL – 3581/LN

### Reagents

All applied reagents were of analytical grade. For the preparation of all solutions, demineralised water (Millipore) was applied. Ultrapure demineralised water had been obtained by Milli-Q water purification system (Millipore, Bedford, MA, USA). Concentrated nitric acid (HNO_3_, 65%) from Merck (SupraPur, Darmstadt, Germany) was applied. The purge gas was argon at purity 99.99%. The certified reference material (Corn Flour, INCT-CF-3) was purchased from the Institute of Nuclear Chemistry and Technology—Department of Analytical Chemistry (Warsaw, Poland).

### Instrumentation

To minimise any potential impurities from other sources, all steps during the sampling procedure were carried out in plastic equipment. Laboratory glasswares (volumetric flasks, funnels etc.) were kept overnight in a 10% nitric acid (HNO_3_) solution and rinsed with deionised water and air dried before use.

A CEM MDS-2000 microwave digestion system (CEM, Matthews, NC, USA) was applied for the digestion of ointments. All measurements for the determination of Cu, Mn and Zn were carried out using a Perkin-Elmer 5100 ZL spectrometer (Perkin-Elmer, Norwalk, CT, USA; FAAS). Cu, Mn and Zn hollow cathode lamps were used as the emission sources. Argon (99.999%) was applied as a purge gas. Background corrections were performed by Zeeman background correction. Further information about instrumentation and detailed parameters are described in Supplementary Material 1.

### Sample Preparations

Before element determination, each ointment was homogenised. Because most of the ointments had an aluminium lid which could be a potential source of EIs, the first few centimetres of each ointment from the tube was discarded. Of each sample, 0.3 g was weighed, poured into Teflon vessels and digested with 5.0 mL of concentrated nitric acid (HNO_3_, 63%). The closed vessels were microwaved after 2 h. The detailed information about digestion procedure are described briefly in Supplementary Material 1. The samples were later cooled at room temperature (25 °C), and the final volume was made to 20 mL. The cooled samples were stored in plastic bottles as stock sample solutions until analysis. This methodology is based on our previously published articles [5, 11]. Five replications were kept and done for all samples to increase the precision of the result.

### The Essential Elemental Impurity Determination and Toxicological Risk Assessment Procedure

**Fig. 1:**
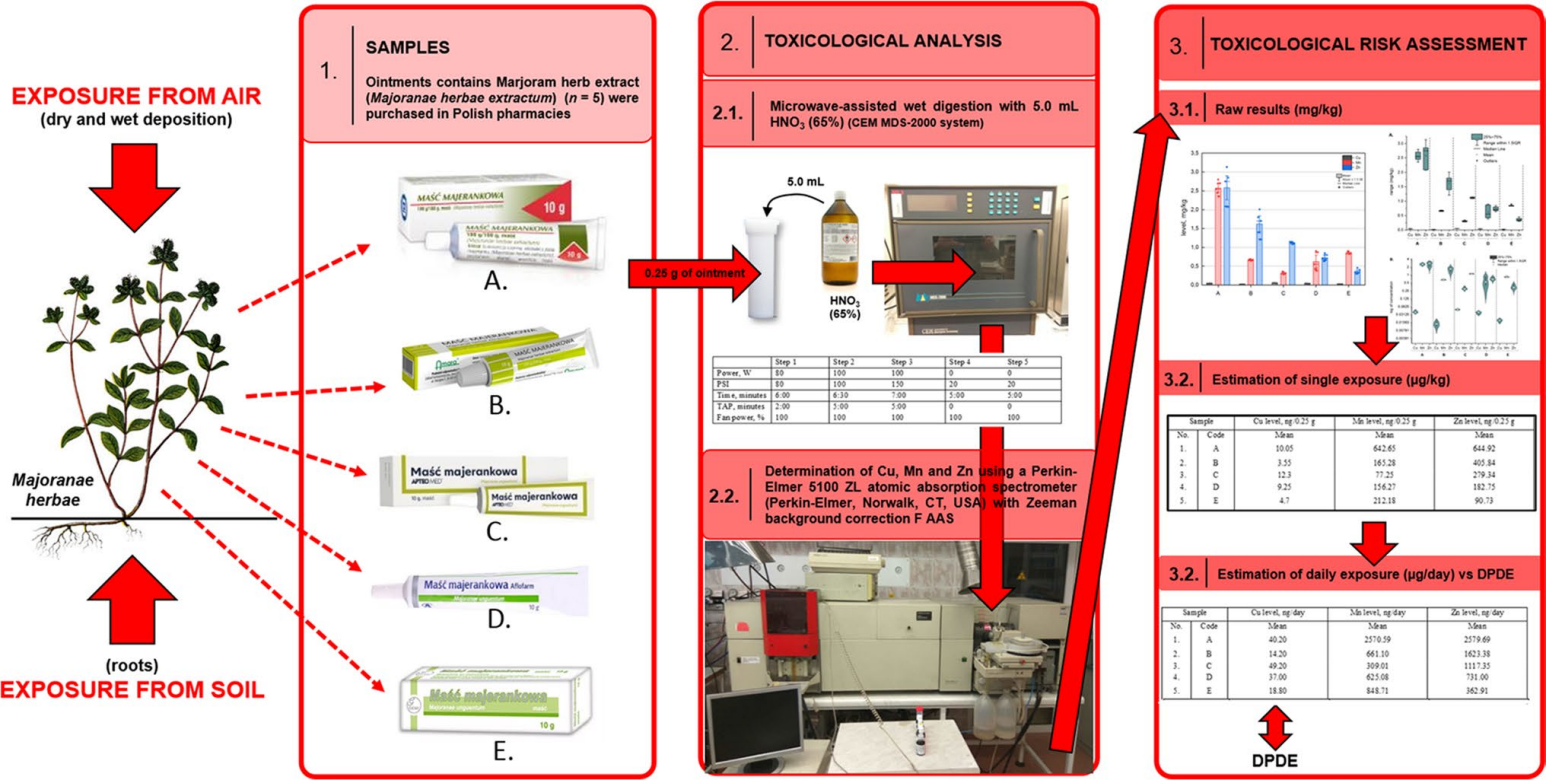
The summary of all health risk assessment steps applied in studies

All elemental impurities were determined in the digested ointment samples using FAAS (described earlier). All instrumentation and detailed parameters for this step are described briefly in Supplementary Material 1. The overview of our toxicological risk assessment has been schematically shown in Fig. 1.

### Analytical Calibration and Quality Control Approaches

In all situations, very good linearity with good correlation coefficients (0.998 for Cu, 0.997 for Mn and 0.999 for Zn) were observed. The values of the correlation coefficients confirm the linearity of the AAS instrument for precision and accuracy of results. Five replications were performed for each sample. Additionally, the quality control and validation of applied methodology are confirmed by previously described studies using the same methodology and apparatus [5, 11]. The synthetic summary of analytical calibration parameters and quality control results are shown in Supplementary Material 1.

### Data Analysis

The results of five independent replicates were expressed as the mean ± standard deviation. Additionally, the descriptive statistics were made (minimum, maximum, mean, skewness and kurtosis) using Origin 2021 Pro licenced by the Jagiellonian University in Krakow. All plots were made using Origin 2021 Pro licenced by the Jagiellonian University in Krakow.

## Results and Discussion

### The Elemental Impurity Profiles of Cu, Mn and Zn in Ointments with Marjoram Herb Extract

**Fig. 2:**
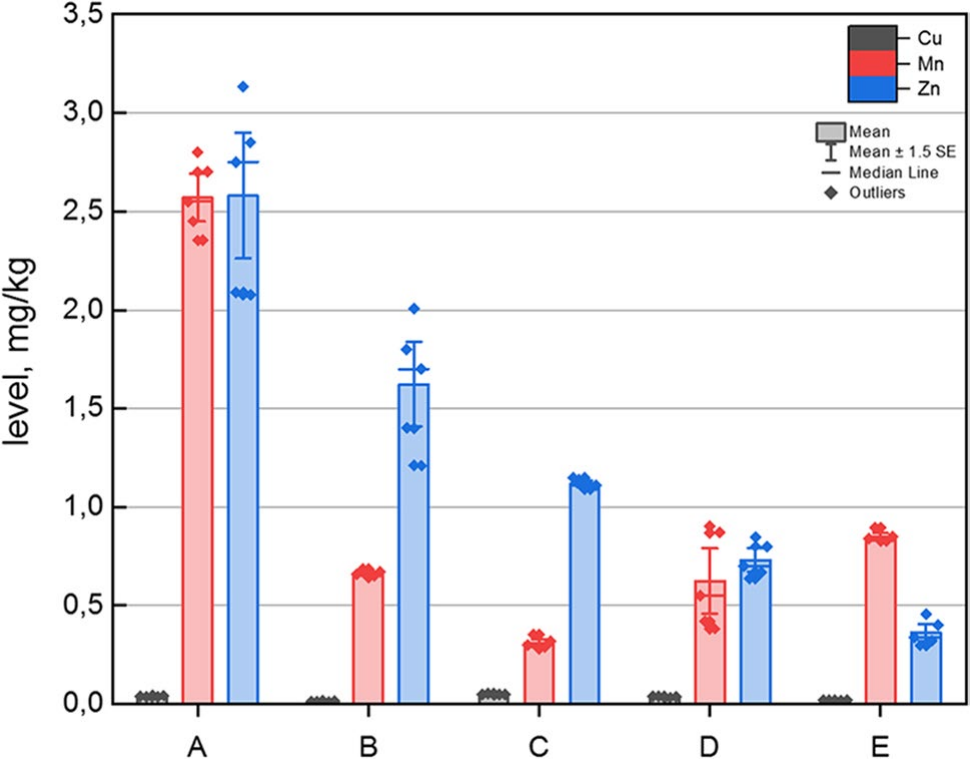
The elemental impurity profile for Cu, Mn and Zn in investigated herbal medicinal products with Marjoram herb extract (A, B, C, D and E)

**Fig. 3:**
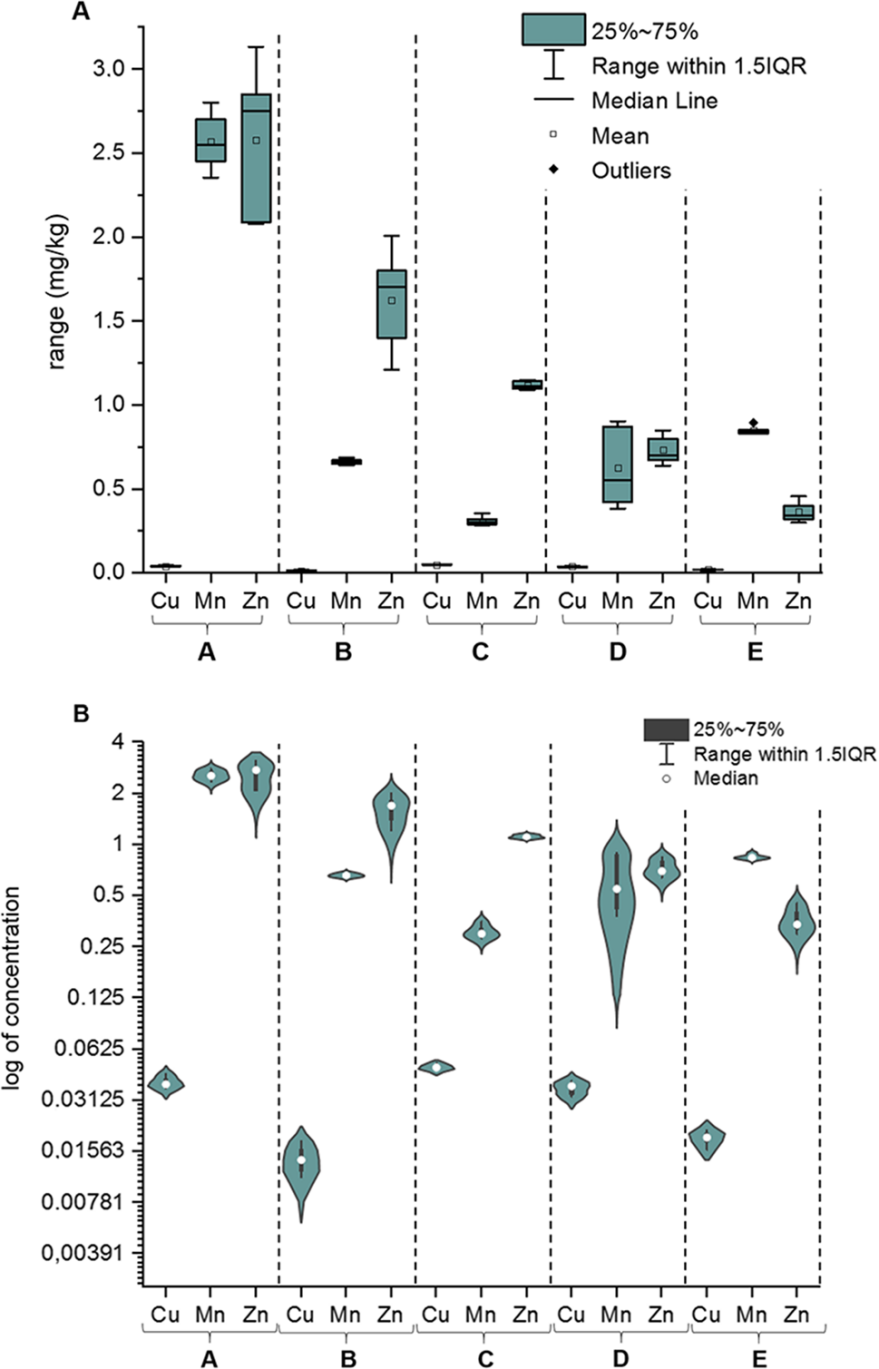
Concentrations of essential elemental impurities (Cu, Mn and Zn) in all investigated herbal medicinal products with Marjoram herb extract (A, B, C, D and E): **A** The boxplot (linear scale). **B** The violin plot showing log_2_ (logarithmic scale) values of concentration

The elemental impurity profiles of analysed samples (*n* = 5; A–E) are presented in Fig. 2, as the graph for each element (mg/kg) determined in analysed HMPs available in Polish pharmacies. Additionally, the boxplot showing ranges of levels and violin plot showing log2 levels of all investigated elements are shown in Fig. 3A and B, respectively. The descriptive statistics of Cu, Mn and Zn contents in all samples is briefly described in Table 2.

**Table 2:** The descriptive statistics of Cu, Mn and Zn levels in investigated herbal medicinal products with Marjoram herb extract available in Polish pharmacies

Element	Minimum, mg/kg	Maximum, mg/kg	Mean, mg/kg	Skewness	Kurtosis
Cu	0.0142	0.0492	0.0319	1.12	1.75
Mn	0.309	2.570	1.003	0.89	1.95
Zn	0.363	2.580	1.283	0.94	1.89

In general, all investigated elemental impurities were present in all analysed samples below 3.5 mg/kg (in the range of 0.0142 to 3.19 mg/L).

The level of copper is extremely lower than other metals. The violin plot (Fig. 3B) showing log_2_ (concentration) values of indicate that level of copper is similar in all investigated samples (in the range of 0.0142 to 0.0492 mg/kg). On the other hand, the Mn level was different (in the range of 0.309 to 2.579 mg/kg; Fig. 3A). Additionally, the Zn level was also different (in the range of 0.363 to 3.19 mg/kg; Fig. 3A). The descriptive analysis of the overall content (Table 2) shows that Cu levels (mean = 0.032 mg/kg) were approximately 31 times lower than Mn levels (mean = 1.0029 mg/kg), and Cu content was approximately 40 times lower than Zn levels (mean = 1.283 mg/kg). Additionally, the levels of Mn and Zn were quite similar. The values of skewness and kurtosis confirm the distribution of results and their consistency.

Individual analysis of the content shows the lowest level of copper was in sample B (0.0142 ± 0.007 mg/kg) and the highest level was in sample C (approximately 0.0492 ± 0.006 mg/kg). The lowest level of manganese was in sample C (0.309 ± 0.06 mg/kg), and the highest content was in sample A (2.57 ± 0.06 mg/kg). Finally, the lowest level of Zn was in sample E (0.363 ± 0.07 mg/L), and the highest level was in sample A (3.19 ± 0.09 mg/L).

Considering the levels of limits for Cu in pharmaceutical products via oral route recommended by ICH Q3D guideline (30.0 μg/g [12, 13]), all of the investigated herbal medicinal products with Marjoram herb extract meet the requirements in the guideline. Hence, our results confirm the safety of Cu contents in all samples. On the other hand, manganese and zinc are classified by ICH Q3D guideline as other metals, i.e. ‘elemental impurities for which PDEs have not been established due to their low inherent toxicity and/or differences in regional regulations which are not addressed in this guideline’ [12, 13]. Therefore, required sources of information about the acceptable levels of these elemental impurities should be other guidelines and/or reginal documents like regulations. However, based on literature review, there is a lack of guidelines and/or regional regulations or other related documents about copper and manganese impurities in pharmaceuticals. Therefore, it is not possible to compare obtained values with any existing regulatory documents. Based on scientific literature review, only Sazakli et al. [14] described Mn levels in *O. majorana*, L. leaves (46.48–77.32 µg/g); however, we analysed the final pharmaceutical product (diluted); hence, it is not possible to compare our results with these values. Notwithstanding, it can be summarised that the contents of all impurities are at a very low level (< 3.5 mg/kg).

### Estimation of Exposure of Elemental Impurities (Cu, Mn and Zn) in Ointments with Marjoram Herb Extract Available in Polish Pharmacies

Appropriate toxicological risk assessment of the investigated essential elemental impurities in this kind of pharmaceuticals should include two steps (Fig. 1):

1. Estimation of single exposure (single dose) (µg/kg)
2. Estimation of daily exposure (µg/day) and comparison with cutaneous permitted daily exposure (μg/day)

The required information in this situation is the actual level in the single dose of the product (approximately 0.25 g). The estimated levels of copper, manganese and zinc in the one-time administration of applied ointments are presented in Table 3.

**Table 3:** The levels of Cu, Mn and Zn in analysed samples (ointment, ng/0.25 g) including single dose

Sample		Cu level, ng/0.25 g	Mn level, ng/0.25 g	Zn level, ng/0.25 g
No	Code			
1	A	10.05	642.65	644.92
2	B	3.55	165.28	405.84
3	C	12.3	77.25	279.34
4	D	9.25	156.27	182.75
5	E	4.7	212.18	90.73

This step of calculation is necessary for the next step of toxicological risk assessment, i.e. the daily dermal exposure of investigated elements (the maximum daily dose of applied pharmaceuticals). For this purpose, the data about frequency of use is required. Based on information in the leaflet for each ointment and information from assessment report on *O. majorana* L. from EMA [1], small amount of the ointment should be spread around the nostrils, two to four times daily. Based on this information, the estimated daily exposures to Cu, Mn and Zn through applied ointments were calculated considering the maximum use during the day (Table 4).

**Table 4:** The estimated daily exposure of investigated elemental impurities in analysed ointment (ng/day)

Sample		Cu level, ng/day	Mn level, ng/day	Zn level, ng/day
No	Code			
1	A	40.20	2570.59	2579.69
2	B	14.20	661.10	1623.38
3	C	49.20	309.01	1117.35
4	D	37.00	625.08	731.00
5	E	18.80	848.71	362.91

Table 4 shows that the estimated exposure of Cu levels in five samples is quite similar (14.20–40.20 ng/day). Also, the estimated exposure for Zn is similar (1623.38–2579.69 ng/day). On the other hand, exposure to Mn has been estimated to be variable for these samples (661.10–2570.59 ng/day).

The appropriate toxicological risk assessment is based on comparison of the obtained results with the cutaneous PDE. For this purpose, the generic and conservative approach has been applied for elemental impurities by ICH Q3D. This approach is based on a systematic adjustment of the parenteral PDE, which assumed 100% bioavailability, to derive a cutaneous permitted daily exposure by using a cutaneous modifying factor (in most cases, intact/irritated skin 10; 100%/10% = 10) (Eq. 1) [13]:

1 $$\mathrm{CPDE}=\mathrm{Parenteral PDE}\times \mathrm{CMF}$$

where:

CPDE is the cutaneous permitted daily exposure.

Parenteral PDE is the parenteral permitted daily exposure.

CMF is the cutaneous modifying factor.

Hence, the calculations of CPDE are only possible for copper (manganese and zinc are classified by ICH Q3D guideline as other metals, see earlier section) (Table 5).

**Table 5:** The calculations of cutaneous permitted daily exposure (CPDE) for copper impurities

	Cu
Parenteral PDE (µg/day)	300
CMF	10
CPDE (µg/day)	3000

Acronyms: *PDE*, permitted daily exposure; *CMF*, cutaneous modifying factor; *CPDE*, cutaneous permitted daily exposure.

The applied toxicological risk assessment approach confirms that the estimated dermal Cu daily exposure (μg/day) is below the CPDE value for this element (< 3000 μg/day) in all investigated herbal-based pharmaceutical products. Hence, the applied toxicological risk assessment confirms safety of investigated herbal medicinal product with Marjoram herb extract due to estimated daily exposure of this element.

## Conclusions and Recommendations

To the best of our knowledge, the study about Cu, Mn and Zn contents in herbal medicinal products with *Majoranae herbae extractum* is described for the first time. The contents of Cu, Mn and Zn as impurities in all ointments with Marjoram herb extract (*Majoranae herbae extractum*) available in Polish pharmacies are at a very low level (< 3.5 mg/kg). The toxicological risk assessment approach confirms that the estimated dermal daily exposure of investigated metals is very low. The estimated dermal daily exposure for Cu in comparison to the CPDE in all products is far below established the EMA requirements. It is not possible to obtain CPDE values for Mn and Zn, but based on estimated daily exposures (< 3.0 µg/day), this exposure is very low. Hence, the obtained results are in accordance with the standards of ICH Q3D guideline. It can be concluded that all analysed products with Marjoram herb extract do not represent a health hazard to the patients. The advantages of our research are the (1) practical methodology and (2) relevance of the obtained results from regulatory toxicology point of view (ICH Q3D elemental impurities guideline for pharmaceutical industry). The disadvantage is the applied technique (FAAS) which is slower and more demanding in comparison to ICP-MS. Based on the review of scientific literature, it would be important to carry out a broader toxicological risk assessment including other important metallic impurities and different herbal medicinal products [6, 15–20].

## Supplementary Information

Below is the link to the electronic supplementary material.

Supplementary file1 (DOCX 21 kb)

## Data Availability

All data generated or analysed during this study are included in this published article and its Supplementary Information file.

## Abbreviations

AAS: Atomic absorption spectrometry
API: Active pharmaceutical ingredient
EI: Elemental impurities
FAAS: Flame atomisation atomic absorption spectrometry
HMP: Herbal medicinal product
ICH Q3D: International Council for Harmonisation of Technical Requirements for Pharmaceuticals for Human Use
PDE: Permitted daily exposure
TRA: Toxicological risk assessment

## Code Availability

Educational Analysis Set SAS® 9 licenced by the Jagiellonian University in Krakow and Origin 2021 Pro licenced by the Jagiellonian University in Krakow.
