# Supplementary material for: The Health Risk Assessment of Essential Elemental Impurities (Cu, Mn and Zn) Through the Dermal Exposure of Herbal Ointment Extracted from Marjoram Herb (Majoranae herbae extractum)
Source: Biol Trace Elem Res. 2021 Jul 31;200(4):1981–7. doi: 10.1007/s12011-021-02842-8 (PMC8854273; doi:10.1007/s12011-021-02842-8)
Supplement: Supplementary file 1 — Supplementary file1 (DOCX 21 kb) [file 12011_2021_2842_MOESM1_ESM.docx]

**Supplementary materials 1. Detailed instrumental parameters**

The samples were digested using microwave digestion system (CEM, Matthews, NC, USA). Concentrated nitric acid (65%) for microwave digestion were of spectroscopic grade from Merck (Darmstadt, Germany). The A microwave-assisted digestion procedure is shown briefly in table S1.

**Table S1.** A microwave-assisted digestion procedure.

|  | Step 1 | Step 2 | Step 3 | Step 4 | Step 5 |
| --- | --- | --- | --- | --- | --- |
| Power, W | 80 | 100 | 100 | 0 | 0 |
| PSI | 80 | 100 | 150 | 20 | 20 |
| Time, minutes | 6:00 | 6:30 | 7:00 | 5:00 | 5:00 |
| TAP, minutes | 2:00 | 5:00 | 5:00 | 0 | 0 |
| Fan power, % | 100 | 100 | 100 | 100 | 100 |

Abbreviations: PSI – pounds-force per square inch; TAP – time at pressure min.

The certified reference material was prepared from corn grown in Poland according to Polish standard PN-A-74205:1997. The material was sieved through the 250 μm nylon sieves and stored in a polyethylene (PE) bag. Approximately 50 kg of sieved corn flour was collected. Examination by optical microscopy revealed that Martin’s diameter of over 98% of particles was below 25 μm. The whole lot of corn flour was then homogenized by mixing for 20 hours in a 110 dm3 PE drum rotated in three directions. Preliminary homogeneity testing by XRF method and final checking of homogeneity by NAA after distribution of the material into containers revealed, that it is sufficiently homogeneous at least for a sample size ≥ 100 mg.

In order to assure the long-term stability, all containers with INCT-CF-3 were sterilized by electron beam radiation. Long-term stability was checked by analyzing concentrations of selected elements in the material stored in the air-conditioned room at 20 °C.

Short-term stability was examined by the determination of concentrations of the selected elements in the bottle stored in the CO2 incubator at 37 °C.

The shelf life of INCT-CF-3 has been established to be 31 December 2015.

The material was certified on the basis of a worldwide interlaboratory comparison, in which 92 laboratories from 19 countries. Analytical uncertainties and stability uncertainties were quantified to arrive at combined uncertainties of the certified values.

The comparison of certified values with measured values of determined elements is shown in Table S2.

**Table S2.** The comparison of certified values with measured values of determined elements.

| concentration, µg/g | certified values | measured values |
| --- | --- | --- |
| Cu | 1.63 ± 0.13 | 1.58 ± 0.18 |
| Mn | 4.98 ± 0.22 | 5.05 ± 0.18 |
| Zn | 20.09 ± 0.76 | 19.65 ± 0.78 |

The linear range of the calibration curve reached from the detection limit up to
0.0; 0.25; 0.5; 1.0; 2.0; 3.0 mg/L for Cu, 0.0; 0.25; 0.5; 1.0; 2.0; 5.0 mg/L for Mn, and 0.0; 0.25; 0.5; 1.0; 2.0; 3.0 for Zn mg/L, respectively. The values of correlation coefficients (R) are a good indicator of the linearity for AAS instrument for precision and accuracy of results, in our studies all correlation coefficients were acceptable (R > 0.998).

The limit of detection (LOD) was defined as (3 SD)/*a*, where SD is the standard deviation corresponding to 10 blank injections and “*a*” is the slope of the calibration function obtained for each microelement. The LODs were determined for Cu, Mn and Zn as 2.1 µg/L,
3.2 µg/L and 2.0 µg/L, respectively. The recoveries obtained were acceptable: 96.8.0 % for Cu, 98.2 % for Mn and 97.6% for Zn.

Blank samples of ultrapure water were prepared applying the same procedure as for the samples to assess possible contamination during the sample preparation and analytical calibration step. All blank levels obtained were negligible. Newly prepared standard stock solutions were serially diluted and used to obtain calibration curves.

The detailed instrumental parameters are shown in table S3.

**Table S3.** Instrumental parameters for the determination of Cu, Mn and Zn.

| Operating parameters | essential trace elements | | |
| --- | --- | --- | --- |
|  | Cu | Mn | Zn |
| Wavelength [nm] | 324.8 | 279.5 | 213.9 |
| Lamp current [mA] | 15 | 15 | 18 |
| Slit width [nm] | 0.7 | 0.7 | 0.7 |
| Optimum working range [µg/kg] | 0.2-2.0 | 0.2-2.0 | 1.0-10.0 |
| Air [ L/min] | 9 | 10 | 10.8 |
| Acetylene [L/mn] | 2 | 2 | 2 |
